# Concepts of Lactate Metabolic Clearance Rate and Lactate Clamp for Metabolic Inquiry: A Mini-Review

**DOI:** 10.3390/nu15143213

**Published:** 2023-07-20

**Authors:** Chi-An W. Emhoff, Laurent A. Messonnier

**Affiliations:** 1 Department of Kinesiology, Saint Mary’s College of California, Moraga, CA 94575, USA; 2 Laboratoire Interuniversitaire de Biologie de la Motricité, Université Savoie Mont Blanc, F-73000 Chambéry, France; laurent.messonnier@univ-smb.fr

**Keywords:** lactate clamp, metabolic clearance rate (MCR), exercise, endurance training, maximal lactate steady state (MLSS), lactate threshold (LT)

## Abstract

Lactate is known to play a central role in the link between glycolytic and mitochondrial oxidative metabolism, as well as to serve as a primary gluconeogenic precursor. Blood lactate concentration is sensitive to the metabolic state of tissues and organs as lactate rates of appearance and disposal/disappearance in the circulation rise and fall in response to physical exercise and other metabolic disturbances. The highest lactate flux rates have been measured during moderate intensity exercise in endurance-trained individuals who exhibit muscular and metabolic adaptations lending to superior oxidative capacity. In contrast, a diminished ability to utilize lactate is associated with poor metabolic fitness. Given these widespread implications in exercise performance and health, we discuss the concept of lactate metabolic clearance rate, which increases at the onset of exercise and, unlike flux rates, reaches a peak just below the power output associated with the maximal lactate steady state. The metabolic clearance rate is determined by both disposal rate and blood concentration, two parameters that are mutually interdependent and thus difficult to parse during steady state exercise studies. We review the evolution of the in vivo lactate clamp methodology to control blood lactate concentration and discuss its application in the investigation of whole-body lactate disposal capacities. In conclusion, we assert that the lactate clamp is a useful research methodology for examining lactate flux, in particular the factors that drive metabolic clearance rate.

## 1. Introduction

In recent decades, the central role of lactate as an intermediate metabolite between glycolysis and mitochondrial respiration has been well articulated in the scientific literature [1,2,3,4]. Lactate is formed by the conversion of pyruvate via the enzyme lactate dehydrogenase (LDH) at the end of glycolysis [5] and is removed from the circulation by gluconeogenic and oxidative cells, tissues, and organs [6,7,8]. Transport across cellular membranes is facilitated by a family of membrane-bound monocarboxylates (MCT), whose flux rate and direction depend upon the diffusion gradients of lactate and hydrogen ions [9,10]. Carbohydrate metabolism under fully aerobic conditions relies on lactate shuttling both within and between cells to fulfill various roles [11], most prominently to provide an energetic substrate for oxidative phosphorylation and to serve as a gluconeogenic precursor [2,12]. Studies have shown that lactate may also act as a signaling molecule in various pathways of metabolic regulation, such as in inhibiting lipolysis and fatty acid oxidation [13,14]. Under fasted and resting conditions, blood lactate concentrations are normally less than 1 mM, and can rise to above 20 mM at maximal exercise in a matter of minutes, making blood lactate a widely used biomarker for exercise physiologists and clinicians to objectively gauge exercise intensity [15]. The shuttling of lactate across diverse tissues can also lead to wide-ranging flux capacities (due to endurance training, for instance) during physical exercise [6,16,17,18]. Not only is augmented lactate metabolism a hallmark adaptation in skeletal muscle following endurance training, lactate exerts feedback inhibition on catecholamine response during exercise [19] and may act as a chemoreceptor that increases the ventilatory response [20,21]. Moreover, following long-term exercise training, changes in LDH activity [22] and MCT expression [23] suggest that muscular and metabolic adaptations to exercise are linked with greater lactate flux [6,16]. These exercise effects may exert beneficial influences on brain function, appetite regulation, breathing, aging, and more [20,21,24,25,26]. Thus, following a brief background of the implications of lactate flux, specifically clearance rate, this mini-review will discuss the lactate clamp as a unique research methodology to provide further insight on whole body metabolic turnover. To gather information for this mini review, we searched the term “lactate clamp” in the PubMed database and included animal and human studies, as well as citing relevant articles (dated 1956 to present) to describe the evolution of the methodology. We also pooled together the human studies to date (1992–2013) that quantified lactate metabolic clearance rate during cycling exercise at the maximal lactate steady state (MLSS).

### 1.1. Background on Lactate Clearance

The shuttling of lactate within and between cells is inextricably linked to the metabolic activity and demands of the internal ecosystem [27,28,29]. Specifically, the ability to clear lactate from the circulation is directly dependent on local blood flow, facilitated diffusion across cellular membranes, and mitochondrial oxidative capacity [9,10,30]. When the latter is limited in capacity, tissues exhibit poor cellular uptake and oxidation of glucose, poor oxidation of fatty acids, and a poor ability to transition between lipid and carbohydrate oxidation, defined as metabolic inflexibility [31,32,33]. Mitochondrial dysfunction is characteristic of diseases related to metabolic syndrome, such as insulin resistance and type 2 diabetes [34], and high mitochondrial capacity is observed in the most metabolically fit populations of endurance athletes [35]. In accordance, lactate clearance has also been reported to be highest in fit, endurance-trained populations [6,16,17], while abnormal lactate metabolism, oftentimes highlighted by high blood lactate concentrations, is an early indication of metabolic disturbances that occur in disease states [36,37]. As such, Krentz et al. [37] reported 35% higher fasting blood lactate in non-obese subjects with impaired glucose tolerance compared to subjects with normal glucose tolerance, and Reaven et al. [36] found that severely non-insulin-dependent diabetic individuals exhibited ~30–100% higher lactate concentrations compared to nondiabetic individuals at any given hour across a 24 h measurement period. In type 2 diabetes mellitus (T2DM), lactate also emerged as a potential candidate biomarker of atherosclerosis and cardiovascular disease, the latter being the primary cause of death in T2DM [38,39]. However, since lactate is simultaneously produced in glycolytic cells and cleared in oxidative and gluconeogenic tissues, simply measuring concentration is inadequate to detect changes in lactate flux. Lactate clearance has been proposed as a proxy for mitochondrial function [32] and, in the cases of blunted clearance, may serve a function in the early detection of the metabolic remodeling associated with disease [40].

In the study of substrate turnover, the term metabolic clearance rate (MCR) is a concept of the rate of substrate disposal/disappearance based on a volume of blood cleared of the substrate per unit of time [41]. For example, lactate MCR (mL·kg^−1^·min^−1^) can be calculated in steady-state conditions by the equation derived from Steele, originally used for measuring whole-body turnover rate of blood glucose during the infusion of a stable isotope tracer [42]:

$$\mathrm{MCR}=R_{d}/\left[\left(C_{1}+C_{2}\right)/2\right]$$

where R_d_ is the rate of disposal/disappearance (mg·kg^−1^·min^−1^) and C_1_ and C_2_ are blood concentrations (mg·L^−1^) at sampling times t_1_ and t_2_, respectively. The conundrum with this concept is that the variables, rate of disposal (R_d_) and concentration (C), are interdependent. This interdependency is illustrated by the fact that a high R_d_ reduces concentration [6,43], and yet concentration reciprocally drives R_d_ [16,44]. Since MCR indicates a blood volume cleared of lactate per minute, the physiological meaning of MCR can be interpreted as the efficiency with which lactate is removed from the circulation. For example, when local blood flow, cellular transport, and utilization of lactate are high compared to its production, then blood concentrations remain low, exemplifying the scenario of high MCR. In contrast, a low MCR may result either from low lactate utilization compared to its production, or from high lactate production and utilization occurring in elevated lactatemia. This latter combination is typically observed close to the maximal lactate steady state (MLSS) or the second lactate threshold (SLT) which corresponds to elevated R_d_ (as well as appearance) and blood lactate concentrations close to 4 mM [16]. Moreover, regardless of the diffusion gradient between blood and muscle, elevated lactate concentrations can reduce the efficiency of transport [9]. Exercise intensity at the MLSS is sufficient to depress MCR compared to a 10% lower relative intensity, which elicits a lower Rd as well as a proportionally lower blood lactate concentration [7,16].

Therefore, the limit of lactate MCR is concurrent with high lactatemia even when the disposal rate is high. In skeletal muscle, the rate of lactate disappearance is greatly dependent on the oxidative capacity of the mitochondrial network, providing a link between lactate uptake capacity and mitochondrial function in muscle. In this context, we review the use of a clamp technique under steady-state conditions, in which blood lactate concentration is held constant, to offer unique insight into whole-body lactate disposal capacities.

### 1.2. The Clamp Technique

Prior to discussing a lactate clamp (i.e., holding blood lactate at a steady concentration above the normal/expected values and/or to a target value), it may be helpful to review the well-established clamp methodologies applied to glucose. Half a century ago, the in vivo glucose clamp technique was described in detail [45,46,47] and it has since played a pivotal role in elucidating the dynamics between glucose and insulin in health and disease. This methodology is generally carried out in one of two ways. The first is to raise the plasma glucose concentration to a fixed hyperglycemic plateau, as in the hyperglycemic clamp. The second is to maintain basal glucose concentration under variable insulin concentrations, as in the hyperinsulinemic euglycemic clamp. In both clamp techniques, steady glucose concentration is maintained for approximately two hours, which requires meticulously adjusting the intravenous glucose infusion rate based on regularly measured blood concentration. Thus, the glucose infusion rate equals whole-body glucose disposal/disappearance rate and provides an indication of β-cell sensitivity and insulin resistance [48]. Combined with an isotope tracer infusion, the clamp technique offers a powerful tool to elucidate metabolite turnover across specific tissues during a prolonged steady-state duration [49,50].

The field’s extensive knowledge of glucose metabolism and insulin sensitivity in healthy and diabetic populations, under various states, including but not limited to rest, exercise, fasted, and postprandial states [51,52,53,54], are due to the plethora of quantitative assessments carried out through these in vivo clamp techniques. Therefore, might a methodology such as an in vivo lactate clamp be as elucidating as the glucose clamp? In a steady-state clamp, lactate infusion rate equals whole body lactate disposal/disappearance, which primarily occurs in skeletal muscle during exercise. Blood lactate concentration can be held at a desired level, possibly affecting multiple systems at once, from skeletal muscle to myocardium, lungs, and brain [28,55,56]. Lactate plays a key role in the energy metabolism of numerous systems, including the inhibition of lipolysis of adipocytes, independent of pH, through the activation of the lactate receptor hydroxycarboxylic acid receptor 1 (HCAR-1), formerly known as the G protein-coupled receptor GPR81 [14]. This example of lactate’s role as a signaling molecule begs the question of how a lactate clamp may be applied in studying whole-body lipid metabolism and the ability to switch utilization between substrates, the very basis for metabolic flexibility, at the onset and cessation of the clamp, for example. This framework of examining metabolic flexibility via a lactate clamp could be expanded to investigate other factors that regulate skeletal muscle substrate utilization, addressing the potential roles of lactate as a signaling molecule [26]. For example, if the shift from fat to carbohydrate utilization during increasing exercise intensity is due to the activation of the pyruvate dehydrogenase complex (PDC) [57,58], and the concomitant lactate-related downregulation of fat utilization via the aforementioned mechanisms and inhibition of carnitine palmitoyltransferase 1 (CPT1) [3], one might develop interesting studies on metabolic flexibility with regard to high-fat dietary intake and simultaneous high lactatemia.

The future possibilities for integrated approaches to study the roles of lactate are truly vast [12], and the clamp technique may offer a unique insight particularly at lower exercise intensities that elicit a steady lactatemia of less than 4 mM. Below, we summarize the evolution of lactate infusions and clamps within the scientific inquiry of substrate metabolism and relevant methodological considerations.

## 2. Previous Studies Utilizing a Lactate Infusion or Clamp

### 2.1. Lactate Infusion or Bolus Studies

Experimental techniques that utilize an in vivo lactate infusion, typically involving a sodium lactate infusate with or without an isotope lactate tracer, have been used for several decades in animals [59,60,61] as well as in humans [7,16,44,62,63,64,65]. Freminet et al. [60] published one of the first “short perfusion” experiments to investigate the role of prolonged exogenous lactate on lactate turnover and oxidation in rodents. Sodium lactate was infused in conjunction with a [U-^14^C]lactate tracer into anesthetized and mechanically ventilated rats for 30 min, beyond which steady high lactatemia could not be sustained. When exogenous lactate raised concentrations from 1.8 mM to 5.8 mM, lactate oxidation as a percentage of total metabolic rate increased from 15% to 45%, respectively, demonstrating the importance of lactate as an oxidative fuel.

With no change in overall metabolic rate, a shift toward increased lactate oxidation must be accompanied by a decrease in other sources of energy. Boyd et al. [62] were the first to utilize a sodium lactate infusion in humans during prolonged submaximal aerobic exercise. Exogenous lactate was infused during the last 30 min of a 90 min steady-state cycling exercise at 40% of maximal oxygen consumption ($\dot{V}$O_2max_), a low exercise intensity that relies primarily on fatty acid oxidation and induces little change in blood lactate concentration in healthy subjects. When lactate was exogenously raised from submaximal exercise levels of 1.0 mM to 8.8 mM, glycerol and free fatty acid concentrations were reduced, demonstrating that high lactatemia inhibits lipolysis. These findings, combined with in vitro studies reporting antilipolytic effects of lactate [13], collectively point toward lactate’s role in metabolic signaling.

Chiolero et al. 1999 [64] carried the procedure further in human subjects by utilizing a sodium lactate bolus infusion over 3 h, during which near steady state was achieved during the third hour of infusion. Continuous measurements permitted the analysis of time course changes in plasma hormone, substrate, and gas exchanges. Increases in the thermic effect of lactate exceeding the theoretical energy cost of oxidation suggested the prevalence of gluconeogenesis as a major metabolic pathway of lactate under resting conditions.

Studying lactate as a function of metabolic rate also raised interesting questions about its role in exercise physiology as a performance indicator. Using three relative work rates (25%, 50%, and 66% of $\dot{V}$O_2max_), Ryan et al. [63], infusing exogenous sodium lactate into healthy human subjects during 20 min of rest followed by 20 min of exercise, reported that a constant infusion rate resulted in varying steady state blood lactate values. Despite a small sample size, these findings demonstrated the important concept that lactate turnover rates are variable depending on exercise intensities, and the responses to exercise are variable across individuals. Since these early studies, multiple reports have successfully quantified lactate production and utilization in energy substrate metabolism [6,43], and with lactate shuttling at its core, giving rise to the concept of the lactate threshold as an individualized indicator of exercise training and endurance performance [66,67,68].

### 2.2. Lactate Clamp Studies

Two decades after the use of a lactate infusion in humans was becoming mainstream, Gao et al. [61] published a study validating the use of an in vivo lactate clamp to assess changes in whole-body lactate disposal in conscious, unstressed rats. Reminiscent of the glucose clamp technique, the significance of this methodology was the ability to maintain a steady lactatemia for a prolonged duration of 90 min by adjusting infusion rates based on concentration measurements, as well as to match the lactate concentration of an experimental condition to determine its effect on metabolism. During the simultaneous infusion of dichloroacetate (DCA), a known enhancer of lactate utilization via the activation of pyruvate dehydrogenase (PDH), the lactate infusion rate had to be markedly increased in order to maintain the same mild hyperlactatemia of 2.0 mM. While important in substantiating the technique, the findings of this study were limited to resting conditions, and the fate of the lactate disposal was not measured.

Experimental studies to apply an in vivo lactate clamp, combined with isotope tracer infusions in exercising humans, were pioneered by the Brooks Lab and first reported on by Miller et al. [69] in a longitudinal endurance training study in healthy men. Two tracers, [6,6-^2^H_2_]glucose (D_2_-glucose) and [1-^13^C]glucose, were simultaneously infused to quantify glucose utilization and recycling during rest and exercise. Under steady state conditions, blood glucose, blood lactate, and expired carbon dioxide were sampled, and isotopic enrichments were measured with a gas chromatography–mass spectrometer using a procedure described previously in detail [69]. For the lactate clamp, an unlabeled sodium lactate–lactic acid cocktail was infused to match the lactate concentration (~4 mM) that was observed during the last hour of a 90 min cycling exercise at 65% of $\dot{V}$O_2peak_ prior to 8–12 weeks of endurance training. After training, the lactate clamp experiment was conducted during rest, as well as during the last hour of a 90 min cycling exercise at the same absolute and relative workloads as the pre-training trial. Due to a training-induced increase in $\dot{V}$O_2peak_, the post-training trial at the same absolute workload corresponded to 55% of $\dot{V}$O_2peak_. In this elegant study design, Miller et al. [69] found that exogenous lactate infusion raising lactatemia to 4 mM during rest decreased the glucose oxidation from ~30% to ~20% of total glucose disposal. Importantly, this glucose sparing effect extended into exercise up to a submaximal intensity corresponding to 55% of $\dot{V}$O_2peak_, where clamping lactatemia at 4 mM decreased glucose oxidation from ~95% to ~75% of total glucose disposal, with no significant change in fuel partitioning. The benefit of lactate loading to provide an energetic substrate appeared to diminish as exercise intensity approached 65% of $\dot{V}$O_2peak_; however, at this intensity, the exogenous infusion of lactate was necessarily low or ceased at some points.

A subsequent cross-sectional study by the same group repeated the comparison of prolonged exercise at 55% and 65% of $\dot{V}$O_2peak_ in healthy men using isotope tracers D_2_-glucose and [3-^13^C]lactate to quantify glucose and lactate kinetics at rest and during exercise [44]. The lactate clamp was performed as carried out in their previous study, matching lactate concentration at rest and during exercise at 55% of $\dot{V}$O_2peak_ to that measured in the 65% of $\dot{V}$O_2peak_ exercise trial. While this study did not include a training component, it was suggested that the low infusion rate of lactate at the higher exercise intensity was necessary due to a significant reduction in metabolic clearance rate of lactate, measured as the rate of disposal relative to blood concentration. Through these two studies, Miller et al. [44,69] demonstrated that a physiological capacity for metabolic clearance may be the driver for the onset of blood lactate accumulation at the lactate threshold, highlighting the importance of understanding the lactate shuttle within and between cells that contribute to the production and utilization of lactate during exercise.

Furthering this investigation in glucose and lactate kinetics as they relate to endurance training and exercise at the lactate threshold, Messonnier et al. [16] most recently implemented an in vivo lactate clamp study during 60 min of steady-state exercise at (or nearest to) the second lactate threshold (SLT) in highly endurance-trained male cyclists. The same two tracers, D_2_-glucose and [3-^13^C]lactate, were simultaneously infused to allow measurements in glucose and lactate kinetics during rest and exercise. An unlabeled sodium lactate infusion was applied to implement a workload 10% lower than LT, while clamping lactatemia at LT concentration (4.3 mM). During exercise at LT, the trained cyclists maintained substantially higher absolute workloads and relative intensities (260 W and 75% $\dot{V}$O_2peak_) compared to the untrained men of the same study (160 W and 67% $\dot{V}$O_2peak_) and subjects from the previous lactate clamp study (181 W and 65% $\dot{V}$O_2peak_) by Miller et al. [44], thus demonstrating the greatest values of lactate flux ever reported during prolonged exercise. With no significant effect on fuel partitioning between carbohydrates and lipids during exercise, exogenous lactate infusion increased lactate oxidation significantly, with the greatest effect being on muscle glycogen sparing [7].

Considering that lactate disposal occurs in many tissues, another key finding by these lactate clamp studies is that while lactate is quantitatively the most important gluconeogenic precursor [70], a continuous infusion of exogenous lactate does not significantly alter the rates of glucose appearance at the LT intensities studied [8,69], suggesting that hepatic glucose production in healthy overnight-fasted men is not substrate-limited under these conditions. However, providing exogenous lactate does shift the source of glucose production toward lactate-incorporated gluconeogenesis instead of hepatic glycogenolysis [8,44]. All of these aforementioned studies collectively represent the tip of the proverbial iceberg in which a lactate clamp has provided useful insight into substrate metabolism in the context of exercise physiology. We therefore emphasize the potential for the broader applications of the lactate clamp methodology in, for example, clinical or at-risk populations to further understand the relationship between disease states and altered intermediary metabolism.

## 3. Metabolic Clearance Rate Limits Exercise Intensity at Maximal Lactate Steady State

Confirming previous findings [6,17,71], Messonnier et al. [15] observed in cyclists that endurance training decreases blood lactate concentration and increases lactate metabolic clearance rate, but disappearance rate remains unchanged compared to untrained men for a given relative exercise intensity [6,17]. Under natural exercise conditions, it is impossible to isolate the effect of training per se on the capacity for lactate rate of disappearance, due to the reduced lactatemia at the same relative work rate after training, as high concentrations may “push” lactate disposal [44]. To address the complexity of the interdependence between rate of disappearance and blood concentration, the lactate clamp procedure allows a comparison between trained and untrained subjects at the same relative work rate and at the same blood lactate concentrations. Indeed, when the lactate clamp matched concentrations in trained and untrained individuals at the same relative exercise intensity of ~65% of $\dot{V}$O_2max_, the rate of lactate disappearance in the trained group significantly increased by nearly 60%, even though metabolic clearance rate decreased by 17% [16]. The fates of this increased lactate removal included primarily oxidation and to a lesser extent gluconeogenesis [7,8,18]; however, the decline of MCR revealed a limit to which lactatemia drives disposal. Interestingly, a positive correlation can be observed between oxygen uptake and MCR at (or near) the maximal lactate steady state. This correlation reinforces the idea that MCR delineates/limits exercise intensity at maximal lactate steady state (MLSS).

**Figure 1:**
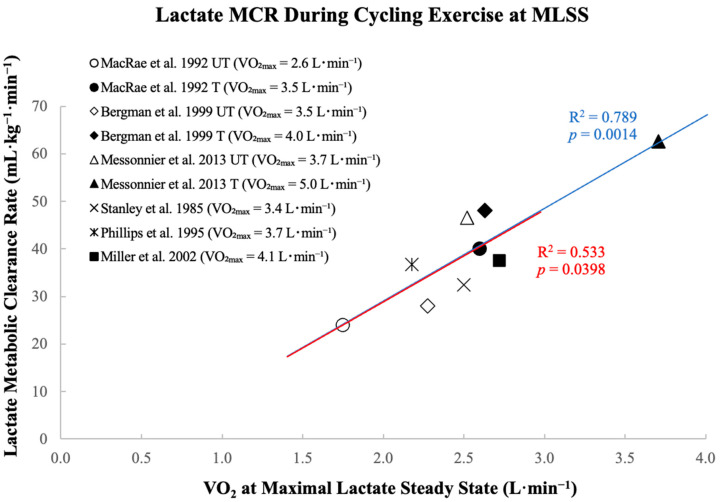
Lactate metabolic clearance rate (MCR) is positively correlated with oxygen consumption ($\dot{V}$O_2_) corresponding to cycling exercise at maximal lactate steady state (MLSS) for cited studies (blue trendline) [6,16,17,43,44], including when the highest point is removed (red trendline). MCR and $\dot{V}$O_2_ values are means from respective studies, some including UT (untrained) and T (trained) subjects.

During a graded exercise test, the lactate rate of disappearance increases with workload, but the metabolic clearance rate rises at the onset of exercise and drops at the highest intensities [17,43]. Similar observations have been made during steady-state exercises, as lactate MCR at 65% of $\dot{V}$O_2max_, or at the power output corresponding to SLT, was lower than at 45% of $\dot{V}$O_2max_ or at SLT-10%, respectively [6,16]. In aggregate studies including sedentary, active, very active, and endurance-trained men [6,16,17,43,44,71], it is shown that during cycling exercise, $\dot{V}$O_2_ at MLSS is positively correlated with lactate MCR (Figure 1), albeit not necessarily the maximal MCR, highlighting a hallmark training effect on lactate metabolism. Even when the highest MCR point is removed, the correlation remains the same. The observation that lactate MCR increases with endurance training, and declines as cycling intensity approaches the highest workload at which lactate steady state can be maintained [16], further corroborates the conclusions from Miller et al. [44] that an individual’s lactate threshold may be determined by a limitation in metabolic clearance rate. Whether this relationship exists in female endurance athletes and other populations with distinct metabolic profiles, and how lactate MCR is linked to health and performance metrics, are questions that warrant further investigation.

## 4. Methodological Considerations with the Lactate Clamp

The practice of combining sodium lactate/lactic acid cocktail infusions with isotope tracers is most useful in quantifying the role that lactate plays in whole-body carbohydrate metabolism, as it allows the quantification of rates of appearance of, disposal of, mitochondrial oxidation of, and conversion to glucose. This methodology of simultaneously infusing labeled and unlabeled substrates is a significant undertaking given the increased cost and complexities of implementing isotope tracer studies in vivo. Additionally, the multitude of downstream fates of a labeled lactate tracer, including conversion to tricarboxylic acid cycle intermediates [4,72], gluconeogenesis to glucose or glycogen, or irreversible disposal via oxidation to CO_2_, complicates the calculations of lactate utilization and typically requires a second tracer, such as D_2_-glucose, to quantify turnover [8,16,44,69]. Without simultaneous tracers during whole body infusions, it is impossible to draw conclusions about tissue-specific flux since cell–cell lactate shuttling occurs throughout the body.

Many reports have documented the mild effect of the intravenous infusion of sodium lactate on the acid–base balance by increasing the strong ion difference and thereby also the buffering capacity of the blood [64,73]. On resting subjects, a hypertonic sodium lactate infusion to raise resting blood lactate concentrations to ~4 mM has been reported to result in a decrease in pulmonary respiratory exchange ratio (RER) values due to a slight increase in $\dot{V}$O_2_ and a slight decrease in $\dot{V}$CO_2_ [8,69], thus underestimating the carbohydrate oxidation. Linked to the peripheral chemoreceptor drive, pulmonary ventilation is also observed to decrease during sodium lactate infusion, although rarely achieving significance [8,69,73]. During exercise, the lower infusion rate needed, and the increased cotransporter activity of MCTs moving lactate and H^+^ protons across plasma membranes, reduce any changes in acid–base variables caused by the lactate clamp, becoming negligible at higher exercise intensities [73]. Also observed during a lactate clamp is a drop in hematocrit, suggesting an increase in plasma volume due to the infusion, but not enough to significantly alter heart rate or mean arterial pressure [8,69].

Further methodological considerations include the point that a prolonged lactate clamp provides energy, which can be a considerable nuance during glycogen-depleting endurance exercise, or an interesting tactic in nutritional support studies. Recent studies in rodents have reported the effects of exogenous lactate administration on elevated carbohydrate and fat metabolism [74,75], suggesting the potential for a lactate nutritional supplement. However, any comparison between oral administration and an intravenous infusion/clamp must discuss the fact that the former involves the passage of lactate through the liver, an organ that is highly active in lactate metabolism. As such, within the context of a lactate clamp providing a carbon energy source, the thermic effect of sodium lactate infusions has been reported and explained as an augmentation in gluconeogenic activity [64].

As is the case with most infusion studies, adequate time is needed to precede measurements with a priming phase, and changes in extracellular fluid volume must be taken into account, all of which have been thoroughly documented in previous investigations [42,73]. Additionally, because of the diverse opportunities for lactate turnover, maintaining a constant blood concentration to achieve the clamp may require delicate practice. Lastly, changes in lactatemia may stimulate many systems at once, making it difficult to tease out specific mechanisms.

Despite the aforementioned methodological challenges, unlike glucose or insulin that have life-threatening lower and upper limits of circulating concentrations, blood lactate benignly exists across a large concentration range, reducing the risk for under- or over-infusion rates during a lactate clamp procedure. Furthermore, studies do not require the simultaneous infusion of insulin, somatostatin, or other counterregulatory hormones. The ideal condition is steady-state low-intensity exercise when lactate turnover is naturally elevated and sustained. Conveniently, there are no documented effects of lactate infusion on the rating of perceived exertion during exercise [8,69], and low-intensity exercise can be safely achieved by populations of all cardiorespiratory capacities.

## 5. Conclusions

The ubiquitous role of lactate in various physiological functions in health, performance, and disease, presents an opportunity to develop creative and multidisciplinary scientific inquiries that build upon current methodologies. The possibility for interdisciplinary inquiries across diverse populations is particularly exciting. Implications related to metabolic clearance rate may provide insight on mitochondrial function at various levels of steady state, further inspiring study designs for the scientific inquiry of metabolic disturbances in pathophysiology. In summary, an in vivo lactate clamp procedure, whether conducted at rest or exercise, is a useful research methodology to conduct metabolic inquiries to elucidate the many roles of lactate in health and disease.

## Data Availability

The data presented in this review are available in the MedLine database.
